# Energy–water and seasonal variations in climate underlie the spatial distribution patterns of gymnosperm species richness in China

**DOI:** 10.1002/ece3.6639

**Published:** 2020-08-08

**Authors:** Bikram Pandey, Janak R. Khatiwada, Lin Zhang, Kaiwen Pan, Mohammed A. Dakhil, Qinli Xiong, Ram Kailash P. Yadav, Mohan Siwakoti, Akash Tariq, Olusanya Abiodun Olatunji, Meta Francis Justine, Xiaogang Wu, Xiaoming Sun, Ziyan Liao, Zebene Tadesse Negesse

**Affiliations:** ^1^ CAS Key Laboratory of Mountain Ecological Restoration and Bioresource Utilization & Ecological Restoration Biodiversity Conservation Key Laboratory of Sichuan Province Chengdu Institute of Biology Chinese Academy of Sciences Chengdu China; ^2^ University of Chinese Academy of Sciences Beijing China; ^3^ Department of Herpetology Chengdu Institute of Biology Chengdu China; ^4^ Botany and Microbiology Department Faculty of Science Helwan University Cairo Egypt; ^5^ Central Department of Botany Tribhuvan University Kathmandu Nepal; ^6^ State Key Laboratory of Desert and Oasis Ecology Xinjiang Institute of Ecology and Geography Chinese Academy of Sciences Urumqi China; ^7^ Xinjiang Desert Plant Roots Ecology and Vegetation Restoration Laboratory Xinjiang Institute of Ecology and Geography Chinese Academy of Sciences Urumqi China; ^8^ Cele National Station of Observation and Research for Desert‐Grassland Ecosystems Cele China; ^9^ College of Geographical Science Fujian Normal University Fuzhou China

**Keywords:** endemic, environmental gradients, gymnosperm richness, human‐induced effects, negative binomial regression, variation partitioning

## Abstract

Studying the pattern of species richness is crucial in understanding the diversity and distribution of organisms in the earth. Climate and human influences are the major driving factors that directly influence the large‐scale distributions of plant species, including gymnosperms. Understanding how gymnosperms respond to climate, topography, and human‐induced changes is useful in predicting the impacts of global change. Here, we attempt to evaluate how climatic and human‐induced processes could affect the spatial richness patterns of gymnosperms in China. Initially, we divided a map of the country into grid cells of 50 × 50 km^2^ spatial resolution and plotted the geographical coordinate distribution occurrence of 236 native gymnosperm taxa. The gymnosperm taxa were separated into three response variables: (a) all species, (b) endemic species, and (c) nonendemic species, based on their distribution. The species richness patterns of these response variables to four predictor sets were also evaluated: (a) energy–water, (b) climatic seasonality, (c) habitat heterogeneity, and (d) human influences. We performed generalized linear models (GLMs) and variation partitioning analyses to determine the effect of predictors on spatial richness patterns. The results showed that the distribution pattern of species richness was highest in the southwestern mountainous area and Taiwan in China. We found a significant relationship between the predictor variable set and species richness pattern. Further, our findings provide evidence that climatic seasonality is the most important factor in explaining distinct fractions of variations in the species richness patterns of all studied response variables. Moreover, it was found that energy–water was the best predictor set to determine the richness pattern of all species and endemic species, while habitat heterogeneity has a better influence on nonendemic species. Therefore, we conclude that with the current climate fluctuations as a result of climate change and increasing human activities, gymnosperms might face a high risk of extinction.

## Graphical abstract

China is one of the global biodiversity hot spot and an important ecological region to study the distribution of gymnosperms including endemic species. Here in this study, we try to evaluate the role of environmental‐ and human‐induced variables in determining the spatial pattern of species richness of gymnosperms. Our findings support that spatial richness pattern of all species of gymnosperms in China is mainly favored by climatic seasonality while energy–water and habitat heterogeneity variables have profound effect on endemic and nonendemic species, respectively.

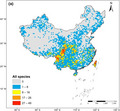

## 1 INTRODUCTION

Species richness patterns are an important topic of research and have been studied for a long time (Currie & Paquin, 1987; Huston, 1979; O'Brien, 1998; Pianka, 1966; Rosenzweig, 1995; Wright, 1983). Ecologists have been trying to determine the impact of various environmental variables that shape the distribution and diversity of organisms in different ecological regions (Fang & Lechowicz, 2006). Explaining the pattern of species richness is a primary goal of ecologists and bio‐geographers (Whittaker, Willis, & Field, 2001) and has gained popularity with the advancement in research and use of modern techniques (Millington, Walsh, & Osborne, 2013). Factors that determine richness patterns are crucial in understanding the structure and dynamics of a population in an area (Currie et al., 2004). Furthermore, recent studies have also prioritized the patterns of species responses to environmental gradients (Pausas & Austin, 2001). Currently, the prediction of richness patterns is more accurate with the use of climatic data generated both from climatic field stations and satellite images (Millington et al., 2013). Studies have also confirmed that not only environmental variables, but also human‐induced effects (e.g., disturbance and agricultural land expansion), are responsible for species richness patterns (Potapov et al., 2017; Stevens, Lehmann, Murphy, & Durigan, 2017; Xu et al., 2019). Previous studies have explained species richness patterns in plants (Dufour, Gadallah, Wagner, Guisan, & Buttler, 2006; Shrestha, Su, Xu, & Wang, 2017; Shrestha et al., 2018) and animals (Keil, Simova, & Hawkins, 2008; Rodrigues, Olalla‐Tárraga, Iverson, Akre, & Diniz‐Filho, 2017), where climatic and human‐induced factors were responsible for determining species richness patterns at the global (Keil et al., 2008; Rodrigues et al., 2017; Sanderson et al., 2002; Shrestha et al., 2018) and regional levels (Dufour et al., 2006; Stevens et al., 2017; Zhao & Fang, 2006). An important goal of these aforementioned studies was to identify the predictors that influence species richness patterns at different spatial scales (Algar, Kerr, & Currie, 2007; Rosenzweig, 1995). Species are not randomly distributed on the earth's surface; instead, they form patterns based on climatic, topographic, and, in recent decades, anthropogenic influences (Currie & Paquin, 1987; Xu et al., 2019). As such, various theories and hypotheses were forwarded to explain the mechanisms governing the distribution of plants and animals (Connell & Orias, 1964; Hawkins et al., 2003; MacArthur & MacArthur, 1961; O'Brien, 1993; Wright, 1983).

Owing to the global loss of biodiversity and subsequent cause of climate change, there is an increasing effort to define the relationship between the number of species and its determinants (Pausas & Austin, 2001). Therefore, this study could be a baseline for predicting biodiversity loss under anthropogenic impacts and future climate change. Moreover, recent studies have prioritized the plant communities belonging to one group—either family or genus—to describe the richness patterns based on environmental, physiological, and biological gradients (Fang & Lechowicz, 2006; Francis & Currie, 2003; Hawkins et al., 2003; Huston, 1979). Therefore, we tried to explore the species richness patterns of gymnosperms in China. Gymnosperms comprise of 1,090 accepted species names reported worldwide (http://www.theplantlist.org/; Forest et al., 2018). China harbors 248 taxa (a compiled list from this study) of gymnosperms, reflecting a global hotspot of gymnosperm species richness. China is also rich in environmental gradients, with tropical to boreal zones, forests to deserts, and high mountains to below‐sea‐level depressions (Xu et al., 2018). The rich diversity of gymnosperms in China might be favored due to the climatic and geographical variation over its vast ecological region (Xu et al., 2018; Zhao & Fang, 2006). Over any large ecological region, the species richness distribution is likely to be driven by two or more environmental gradients (Kreft & Jetz, 2007). Moreover, energy–water, climatic seasonality, habitat heterogeneity, and anthropogenic influence are directly linked to plant diversity and are determinants of regional variation in species richness (Algar et al., 2007; Liu et al., 2017; Shrestha et al., 2017). Therefore, in this study, we tried to evaluate the relative role of these aforementioned factors in explaining the species richness patterns of gymnosperms in China.

The energy–water hypothesis is the most common and highly discussed hypothesis that explains the species richness pattern of an organism (Brown, Gillooly, Allen, Savage, & West, 2004; Hawkins et al., 2003; Keil et al., 2008; Obrien, 2006; Wright, Currie, & Maurer, 1993). This hypothesis proposes that the availability of water and energy determines the total resources available to the plants that control the biological activities, which in turn determines the variations in biodiversity (Adler & Levine, 2007; Jiménez‐Alfaro, Chytrý, Mucina, Grace, & Rejmánek, 2016; Turner, 2004; Wright et al., 1993). Second, the habitat heterogeneity hypothesis is the synergistic association between species distribution and topographic variations (Pausas & Austin, 2001). The existence of environmental or resource heterogeneity may create high niche diversity and allow species to coexist at a large spatial scale (Jiménez‐Alfaro et al., 2016; Kreft & Jetz, 2007; Tamme, Hiiesalu, Laanisto, Szava‐Kovats, & Pärtel, 2010). With the increase in habitat diversity, the species richness also increases and is highly scale‐dependent in a landscape, governing species richness gradients by local and regional species turnover (Dufour et al., 2006; Kerr & Packer, 1997; Kreft & Jetz, 2007; MacArthur & MacArthur, 1961). Third, climatic variability and unsystematic changes in the daily maximum and minimum temperatures increase the tolerance level of an organism by altering the thermal environment that organisms experience, enabling them to become geographically widespread (Chan et al., 2016; Connell & Orias, 1964; Shrestha et al., 2017). Finally, the human‐induced effects such as habitat destruction, habitat fragmentation, land use change, disturbances, and habitat loss, collectively account for limited species distribution (Gambi, Pusceddu, Benedetti‐Cecchi, & Danovaro, 2014; Huston, 1979; Pausas & Austin, 2001). Human activity can directly affect distribution and diversity patterns at the regional scale (Sanderson et al., 2002). Similarly, previous studies have reported the negative impact of human association on the distribution of animals (Fløjgaard, Normand, Skov, & Svenning, 2011; Ilsøe, Kissling, Fjeldså, Sandel, & Svenning, 2017). Gymnosperms are threatened plant species; 40% of these species are at high risk of extinction (Forest et al., 2018; Wu & Raven, 1999). Therefore, studying the impact of human association is also crucial to understanding distribution patterns.

There are studies testing limited hypotheses (Lü, Cai, Yang, Wang, & Zeng, 2018; Lundholm, 2009; Osland et al., 2017; Panda, Behera, Roy, & Biradar, 2017), and multiple hypotheses (Gao & Liu, 2018; Kreft & Jetz, 2007; Liu et al., 2017; Shrestha et al., 2017; Su et al., 2020) to determine the spatial distribution of plant richness. A single variable or hypothesis limitedly explains the richness pattern, as multiple complex phenomena collectively determine the distribution pattern of species richness. Thus, multiple modeling approaches could be most suitable for quantifying the contributions of various hypotheses toward spatial richness patterns. Moreover, there are studies that explain the species richness patterns of gymnosperms along environmental gradients in the Himalayas (Pandey et al., 2020; Subedi, Bhattarai, Perez, & Sah, 2019). Tian (2002), Ying, Chen, and Zhang (2004), Li, Shen, Ying, and Fang (2009), Jiang, Cheng, and Yin (2010), and Yang (2015) have also studied the distribution of gymnosperms in China, while the mechanism that determines richness patterns were not considered. Moreover, the spatial pattern of gymnosperm richness and its relationship with environmental factors in China have been studied (Chen et al., 2013; Lü et al., 2018). The variables used in Lü et al. (2018) may lead to similar species–climate relationships; however, the richness pattern of endemic and nonendemic groups of gymnosperms species in China is little known. Endemic species are confined to specific geographical regions and have restricted distribution, while the distribution of nonendemic species is wide, covering a diverse distribution range (Wu & Raven, 1999). We prioritized differentiating the species of gymnosperms into endemic and nonendemic species with an estimate that the factors affecting the distribution of both endemic and nonendemic species will differ from one another. We tried to explain the spatial richness patterns of endemic gymnosperms based on environmental factors because of the varied climatic conditions in China, which have not been mentioned in previous studies. In contrast, anthropogenic impacts, namely habitat destruction, habitat fragmentation, climate change, and pollution, are suggested to influence the distribution patterns of plants (Xu et al., 2019). To the best of our knowledge, to date, the effect of human influence factors has not been considered when evaluating the spatial richness patterns of gymnosperms species, including endemic species. Therefore, our study will be of great significance for elucidating the driving mechanism of species richness patterns and developing conservation strategies for Chinese gymnosperms plants, especially in the context of a human‐dominated world.

## 2 METHODS

### 2.1 Study area

The study area comprises the geographical region of the Peoples’ Republic of China. China lies within two biogeographic realms (Palearctic and Indo‐Malay) (Olson & Dinerstein, 2002). It consists of five terrestrial biomes: tropical and subtropical moist broadleaf forests, temperate broadleaved and coniferous mixed forests, temperate coniferous forests, temperate broadleaf and mixed forests, and montane grassland and shrublands. These biomes are further divided into 12 ecological regions (Olson & Dinerstein, 2002). These ecological regions are characterized by their rich diversities and endemic species of plants, including gymnosperms (Wu & Raven, 1999). Following previous studies (Liu et al., 2017; Shrestha et al., 2017), we mapped the geographical regions of China using the same projection and overlaid a grid cell of 50 × 50 km^2^ (0.5° × 0.5° at the equator) of spatial resolution. The grid cells with <50% of land cover were excluded, totaling 4,217 remaining grid cells.

### 2.2 Species data

Initially, we prepared the list of all of the native species of gymnosperms recorded from China based on Wu and Raven (1999), Ying et al. (2004), Fang, Wang, and Tang (2011), and Tang (2015), while exotic species were excluded. We followed Wu and Raven (1999) to differentiate the native species from the exotic ones. The list was then validated from the online portal “The Plant List” (http://www.theplantlist.org) for synonyms and nomenclature errors, which restricted the number of gymnosperm taxa (including varieties) to 248. Further, our database consisted of 236 gymnosperm taxa from 248 reported taxa. Spatial distribution occurrence data of 236 gymnosperm taxa were obtained from the National Specimen Information Infrastructure (http://www.nsii.org.cn/; accessed between August 2017 and April 2018), Global Biodiversity Information Facility (https://www.gbif.org/; accessed between November 2017 and February 2018), Chinese Virtual Herbarium (http://www.cvh.ac.cn/; accessed between August 2017 and April 2018), and relevant literature. In this study, we used the geographical distribution occurrences of 236 gymnosperm taxa (184 species and 52 varieties) that represented “all species” distributed within China, including 114 endemic and 122 nonendemic taxa. The endemic taxa were the species or varieties reported from China only. We used the studies of Wu and Raven (1999) and Ying et al. (2004) to differentiate between endemic and nonendemic taxa. Therefore, based on this distribution, we divided the response variables into three groups: (a) all species, (b) endemic species, and (c) nonendemic species. We used the geographical coordinate occurrence of a species to determine its presence or absence in the locality. One may argue that using spatial distribution occurrence will be biased because a species may incorrectly appear to be absent from a particular location if that area has never been surveyed. Ferrier (2002) mentioned that the problem of false absences is less severe when spatial units of analysis are sufficiently large, for example, course grid cells or whole ecological regions. Further, to reduce the sampling bias, following Shrestha et al. (2017) and Liu et al. (2017), we plotted the georeference distribution locations of gymnosperms at the county level. The county‐level distribution maps were then transferred into gridded distributions with a spatial resolution of 50 × 50 km^2^. We overlaid the distributional map of each species with the grid using ArcGIS (v10.3.1) (ESRI Inc.).

### 2.3 Predictive data

The pattern of species richness along environmental gradients has a direct relationship with plant growth and development (Pausas & Austin, 2001). The availability of energy and water can be measured with numerous metrics namely temperature, precipitation, and solar radiation (Evans, Warren, & Gaston, 2005). As a surrogate for available atmospheric energy, we focused on potential evapotranspiration (PET, mm/year) and mean annual temperature (MAT, °C/year), which have been recognized as two of the best predictors of species richness (Evans et al., 2005; Jiménez‐Alfaro et al., 2016; Turner, 2004). Similarly, as a surrogate of water availability, we used actual evapotranspiration (AET, mm/year) and mean annual precipitation (MAP, mm/year) (Jiménez‐Alfaro et al., 2016; Pausas & Austin, 2001). These variables were also used as surrogates for energy and water in determining the species richness of plants in previous studies (Evans et al., 2005; Liu et al., 2017; Lü et al., 2018; Pausas & Austin, 2001; Shrestha et al., 2017). The mean annual PET and AET were downloaded from the MODIS Global Evapotranspiration Project (MOD16, www.ntsg.umt.edu/project/modis/mod16.php). MOD16 used the Penman–Monteith equation to calculate the variables. It has been recommended for large‐scale studies (Mu, Heinsch, Zhao, & Running, 2007). We extracted MAP and MAT from the WorldClim database (http://www.worldclim.org; Hijmans et al., 2005) as variable accounts for temperature (energy) and water availability, respectively (Hijmans, Cameron, Parra, Jones, & Jarvis, 2005). Habitat heterogeneity was measured by the elevation range (ELR, in meters), mean elevation (MElv, in meters), and coefficient of variation in elevation (VEL) as a measure of the roughness of an area. These are the most informative predictors representing habitat heterogeneity and are also used as a surrogate for topographic variations (Jiménez‐Alfaro et al., 2016; Lü et al., 2018; Moura, Villalobos, Costa, & Garcia, 2016; Shrestha et al., 2017). The ELR was calculated as the difference between the maximum and minimum elevation of a grid cell from the data extracted from SRTM 90 digital elevation data (http://srtm.csi.cgiar.org/) (Jiménez‐Alfaro et al., 2016; Shrestha et al., 2017). The MElv values of a grid cells were extracted from the same SRTM in ArcGIS. Furthermore, the annual range of temperature (ART, °C/year), temperature seasonality (TES, °C/year), and precipitation seasonality (PRS, mm/year) were used as surrogates for short‐term climate seasonality (Lü et al., 2018; Shrestha et al., 2017). The ART was measured as the difference between the maximum temperature of the warmest month and the minimum temperature of the coldest month; the TES was the standard deviation of the monthly temperature, and the PRS was the coefficient of variation of the monthly precipitation; all were downloaded from the WorldClim database (http://www.worldclim.org/; Hijmans et al., 2005). Finally, we used the human influence index (HII) and human footprint index (HFI) as proxy variables of anthropogenic impacts that represent human‐induced effects (Sanderson et al., 2002). The HII and HFI were both downloaded from the archives of the Wildlife Conservation Society (http://sedac.ciesin.columbia.edu/data/). HII and HFI data use proxies of human population density, settlements, roads, and other access points to define human influence (Sanderson et al., 2002).

The rasters of variable layers were in different resolutions and projection systems; thus, all the layers of predictor variables were downscaled to the same cell size (50 × 50 km^2^ grid cells) using the resample tool and the same coordinate system using the Project Raster tool in ArcGIS. Further, the mean values of variables in each 50 × 50 km^2^ grid derived from resampling were used for analysis. These datasets of gymnosperm distribution enabled us to predict the species richness patterns based on climatic, environmental, and human‐induced variables. We used the most recently available variables to determine their influences on the richness patterns of gymnosperms in China.

### 2.4 Data analysis

Species richness was defined as the number of species occurring in each grid cell. First, to evaluate the relative importance of environmental variables, based on the objective of our study, we separated the explanatory variables into four distinct predictor sets: (a) energy–water, (b) climatic seasonality, (c) habitat heterogeneity, and (d) human‐induced factors. Because all of the predictor variables were highly correlated, we removed the multicollinearity by performing a principal component analysis (PCA) in each predictor set and extracted the three principal components (Moura et al., 2016). The variance inflation factor (VIF) was used to check for multicollinearity among response variables (Legendre & Legendre, 1998). These PCA components accounted for 96.6% of the variation in energy–water variables, 99.4% in climatic seasonality variables, 95.9% in habitat heterogeneity variables, and 99.9% in human‐induced variables (Table S1). The general rule of thumb is that multicollinearity between predictors is considered to be significant when VIF >5 (Legendre & Legendre, 1998). All variables used in the model had VIF values <5 (see Table S2).

Second, we used generalized linear models (GLMs) to determine the most suitable predictors for explaining the species richness patterns of three response variables (Gambi et al., 2014). The species richness data did not follow a normal distribution and were over‐dispersed (Cameron & Trivedi, 2013). Therefore, following O’hara and Kotze (2010), we did not try to transform the species count data, and performed diagnostic plots of the variance to mean relationship in quasi‐Poisson error distribution and negative binomial regression (NBR) models (Hilbe, 2011). Quasi‐Poisson error distribution and NBR were intensively used for over‐dispersed count data. We selected NBR distribution over quasi‐Poisson (Hilbe, 2011; Zeileis, Kleiber, & Jackman, 2008) as it provided a better description of our data (Figure S3). We followed a variable selection approach to identify the best‐supported model, selecting the predictors by a stepwise backward procedure based on a low Akaike's information criterion (AIC) and high adjusted *R* ^2^ value (Burnham & Anderson, 2003; Legendre & Legendre, 1998). Further, we also assessed the effect of spatial autocorrelation, which could affect our model testing. Therefore, we obtained the model residuals using *Moran's I* values to test for type I error (Legendre & Legendre, 1998). The tests showed a lack of spatial autocorrelation, and thus, spatial autocorrelation was not considered in the models (Figure S4).

Finally, we performed variation partitioning to determine the relative importance of the explanatory power of the best predictor and richness of gymnosperm groups based on the highest variance (Murray & Conner, 2009). This approach allowed us to assess the pure effects of the predictor variables and their shared contribution in better explaining the species richness patterns of gymnosperms. We calculated the proportions of explained deviance for each of the factors included in the GLMs (Liu et al., 2017).

Maps and geospatial products were created using ArcGIS. All statistical analyses were performed using R statistical packages: “MASS” package was used for stepwise regression, “car” package to check VIF of a model, “vegan” package was used for PCA and variation partitioning, and “spdep” was used to perform *Moran's I* test for residual spatial autocorrelation (R Development Core Team, 2017).

## 3 RESULTS

### 3.1 Species richness patterns of all species of gymnosperms

**FIGURE 1:**
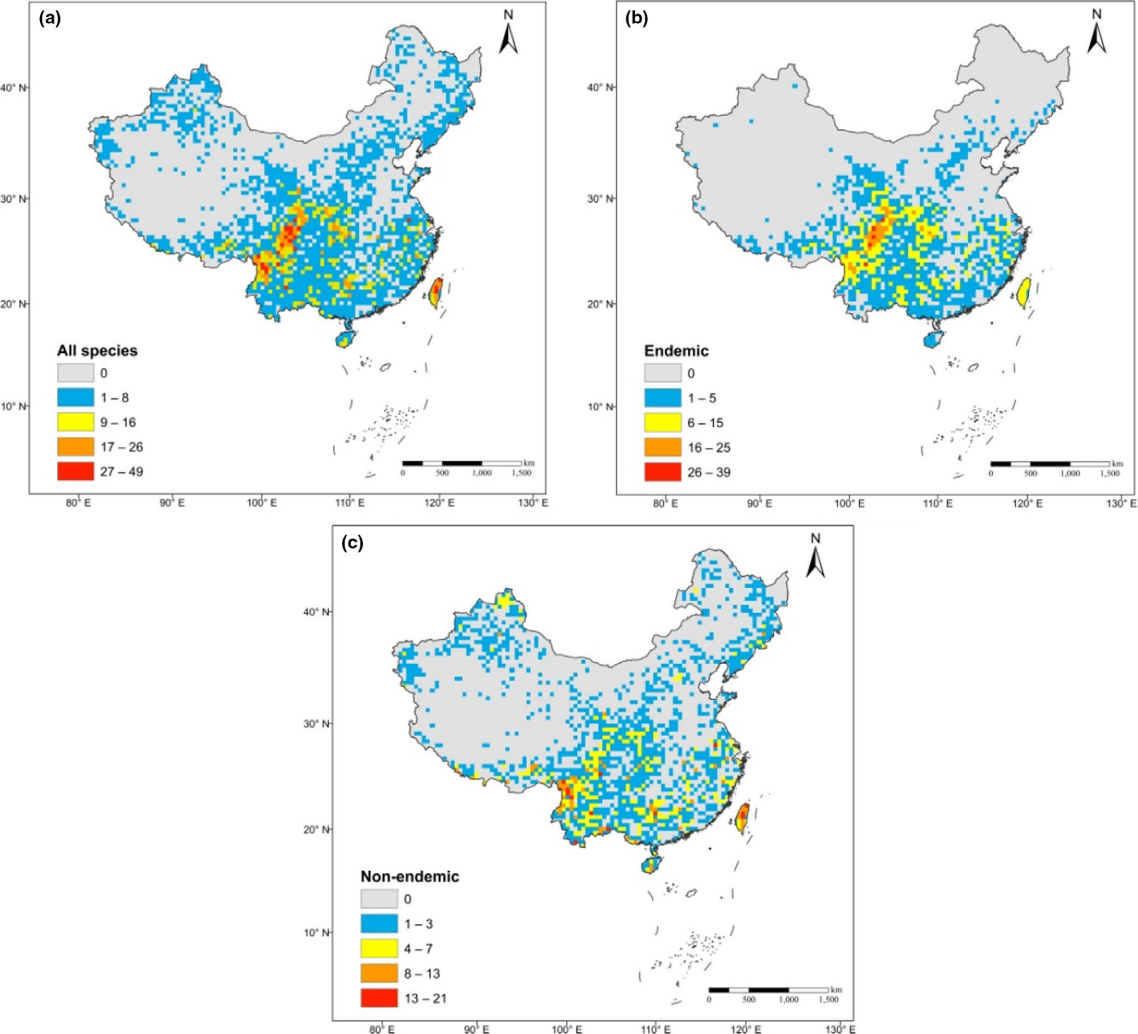
Spatial distribution of gymnosperm species in China across all (a), endemic (b), and nonendemic (c) species. The spatial scale of the grid cell is 50 × 50 km^2^, projected in ArcGIS 10.3.1

All species (*n* = 236) of gymnosperms were found in 1837 grid cells, ranging from 1–49 species per grid cells (mean 4.78 ± 0.13 *SE*) (Figure 1a; Table S5). The distribution of gymnosperms in China is uneven. The southern region of the country (20–30°N and 100–110°E) had the greatest species distribution. Moreover, the island of Taiwan of China (20°N and 120°E) is also characterized by a rich diversity of gymnosperms. However, the distribution of gymnosperms in the northern region of the country is less than that in the southern region.

**FIGURE 2:**
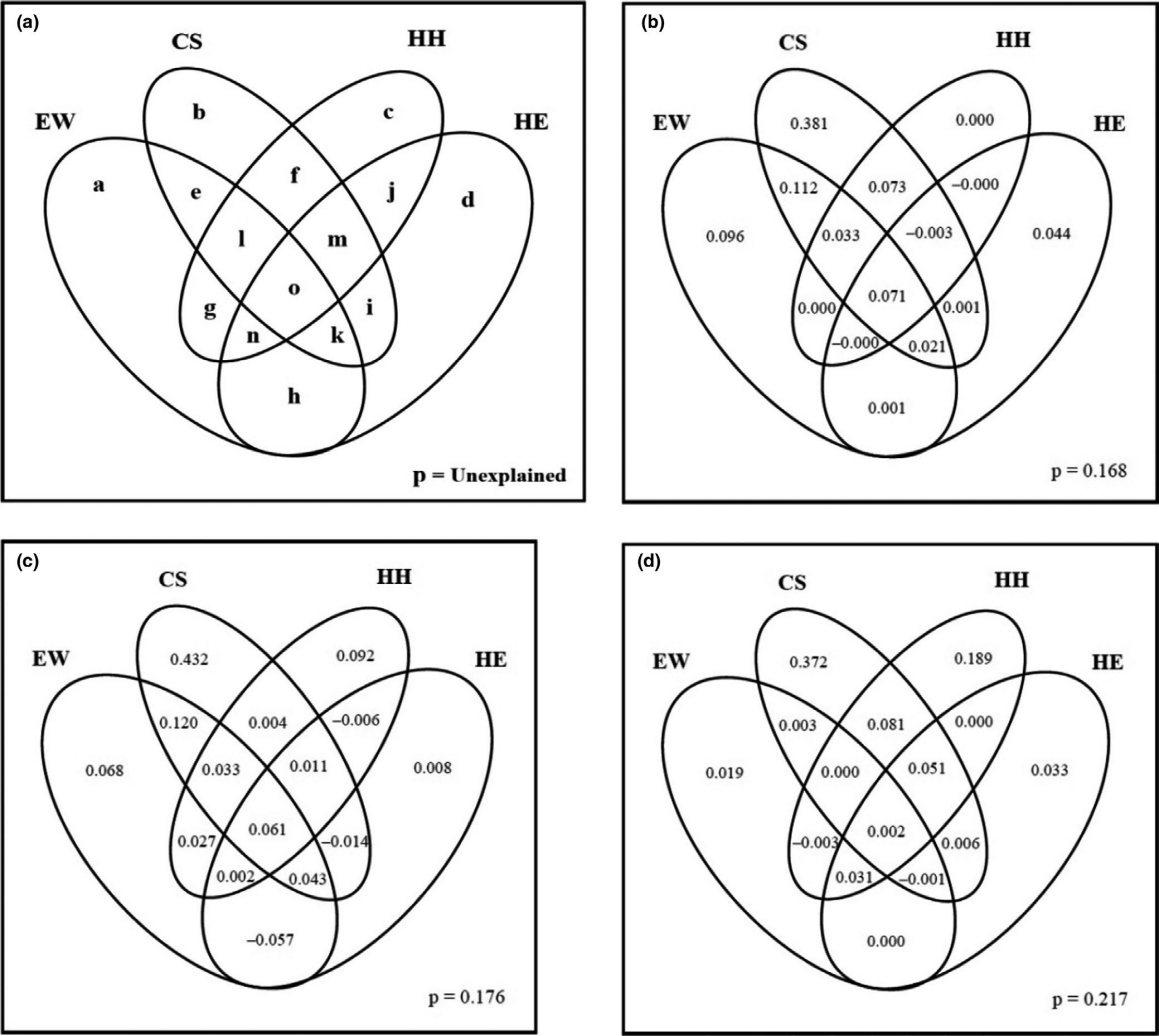
Results of variation partitioning explained by environmental variables (EW = energy–water, CS = climatic seasonality, HH = habitat heterogeneity, HE = human influence effects). (a) Schematic representation of variation partitioning. Each letter in the Venn diagram represents a fraction of variation partitioning analysis. Total variation explained by energy–water [aeghklno], climatic seasonality [befiklmo], habitat heterogeneity [cfgjlmno], and human influence [dhijkmno]. Variation partitioning results for (b) all, (c) endemic, and (d) nonendemic species

With respect to relationship of the predictors set and all species richness, the best model is explained by a set of six variables (CS1 + CS2 + EW2 + EW3 + HH1 + HE3). The significant and higher deviance was explained by climatic seasonality variables (CS1 = 52.46%, *p* < .001; CS2 = 26.64%, *p* < .05) followed by other variables (EW2 = 13.28%, HH1 = 11.6%, EW3 = 9.21% and HE3 = 2.86%) (Table 1). The results of the variation partitioning revealed that the species richness variation explained by all four predictors set was 83.2% for all species. The climatic seasonality predictor set explained 69% variation in all species richness patterns, followed by the energy–water (33.47%), habitat heterogeneity (17.36%), and human‐induced effect predictor sets (13.36%) (Figure 2a,b

; Table S6).

**TABLE 1:** Summary of generalized linear models (GLMs) and the best combination of variables selected after the backward selection method to explain the species richness patterns of gymnosperms in China. “Explained” indicates the percentage (%) of explained deviance. *p*‐values are the significance value of the variable in a model.

	Variables	Z‐value	Explained (%)	*p*‐value
All species	CS1	–19.376	52.46	<.001
	CS2	2.160	26.64	<.05
	EW2	–3.343	13.28	<.001
	HH1	–2.197	11.60	<.01
	EW3	3.806	9.21	<.001
	HE3	–2.631	2.86	<.05
Endemic	CS1	–9.887	41.47	<.001
	EW3	2.852	12.19	<.01
	CS2	3.326	8.17	<.001
	EW2	–2.750	6.46	<.01
Nonendemic	CS1	–14.272	48.71	<.001
	HH1	–4.041	13.02	<.001
	HH2	0.29	1.73	NS
	HE3	0.138	0.63	NS

EW, CS, HH, and HE refer to variables based on the first three axes of the PCA using the energy–water, climatic seasonality, habitat heterogeneity, and human influence effect variables, respectively.

### 3.2 Patterns of endemic species

Endemic species (*n* = 114, about 48% of total species richness) were found in 1,197 grid cells. The endemic species richness ranged from 1–39 (mean 4.18 ± 0.12 *SE*) (Figure 1b, Table S5). Endemic species were mostly concentrated in the southern region of China, while their distribution in the northern part was scarce. Only few taxa of endemic species have adapted to the higher‐latitude environments.

The endemic species richness is explained by four variables grouped into two sets of predictors. The significant and highest explained deviance was shown by CS1 (41.47%, *p* < .001), followed by EW3 (12.19%, *p* < .01), CS2 (8.17%, *p* < .001) and EW2 (6.46%, *p* < .001) (Table 1). In variation partitioning, 82.37% of the variation was explained by all four predictor sets in defining the richness pattern of endemic species. Endemic species richness showed a marked relationship with climatic seasonality predictor set, which explained 68.86% of the variation, followed by energy–water and habitat heterogeneity predictor set explaining 29.68% and 22.46% of the variation, respectively. The human influence predictor set explained the least variation in the richness patterns of endemic species (4.69%) (Figure 2a,c; Table S6).

### 3.3 Nonendemic species and their richness patterns

Nonendemic (*n* = 122) species comprised 52% of the total species richness. They were found in 1,496 grid cells and their distribution ranged from 1–21 (mean 2.6 ± 0.06 *SE*) species in each cell (Figure 1c; Table S5). In contrast to endemic species, the distribution of nonendemic species richness was similar to that of all species of gymnosperms. The southern region and Taiwan of China, harbor high nonendemic species, while the northern region has a lower species distribution.

Finally, with respect to the predictor set and richness patterns of nonendemic species, the final model includes four variables (CS1 + HH1 + HH2 + HE3) showing a varied response. The most significant and highest deviance of nonendemic species richness was explained by CS1 (48.71%, *p* < .001), followed by HH1 (13.02%, *p* < .001). However, there was an insignificant effect of HH2 and HE3 predictors in the model (Table 1). In variation partitioning, 78.24% of the variation was explained by all four predictor sets in determining the richness patterns of endemic species. The highest amount of variation in nonendemic species richness was accounted for the total effect of the climatic seasonality variable set, explaining 51.30% of the variation. Likewise, the variation partitioning also revealed that the habitat heterogeneity and human effect predictor sets followed climatic seasonality, determining 18.89% and 12.16% of the variation in richness patterns of nonendemic species, respectively (Figure 2a,d; Table S6).

## 4 DISCUSSION

China is a global richness center (accounting for almost 23% of the total number of species globally) of gymnosperm species distribution (Byng, 2015; Farjon, 2010), and almost 50% of gymnosperm species are endemic (Ying et al., 2004). Our result indicated that the southwest China and Taiwan province of China had high diversities of gymnosperms, including endemic species. The distribution of the highest endemism might be due the varied ecological regions and variations in climatic conditions that prevail in the southwestern region of China. The climate of southwest China ranges from tropical to temperate and is characterized by high temperature and abundant precipitation (Liu, Chen, Lian, Chen, & Chen, 2015). Moreover, seasonal climatic variation might also favor the diversity of gymnosperms in China (Shrestha et al., 2017; Zhai et al., 1999). In contrast, the extreme and harsh climatic conditions in the north, Tibetan Plateau, and Inner Mongolia regions might be limiting factors for the distribution of gymnosperms (Chen et al., 2013; Zhai et al., 1999), acting as a filter for species without tolerance to climatic factors (Hurlbert & Stegen, 2014). Generally, the results indicated that the border areas of Yunnan, Sichuan, and the Tibet Autonomous Region, especially in the areas of the Longmen, Qionglai, Minshan, Yunling and Gaoligong mountains belonging to the Hengduan Mountain region, have the highest species richness values of all studied groups due to their diverse topographies, hydrothermal conditions, and habitats. These patterns may be explained by combinations of climatic seasonality, energy–water, and habitat heterogeneity in the area (Figure 2). These findings were also supported by Li et al. (2009), Chen et al. (2013), Lü et al. (2018), and Shrestha et al. (2017). Climatic variables are frequently considered to provide strong predictors of broadscale species richness. The influences of climatic seasonality, energy–water and, habitat heterogeneity are significant predictors in explaining distinct fractions of variation in gymnosperm species richness.

The strong relationship between the species richness and climate seasonality, as well as the following variation partitioning results, suggests that climatic seasonality is the most influential predictor variable in describing the species richness patterns among all subgroups of response variables. At coarser spatial resolutions, our measurement of climatic seasonality becomes more important for predicting species richness. We found that climatic seasonality alone is the measure of climatic factors constraining the richness of all, endemic, and nonendemic species of gymnosperms in China. This indicates that the seasonal variation in temperature has a profound effect on species richness (Panda et al., 2017). Previous studies conducted in China (Gao & Liu, 2018; Shrestha et al., 2017) have also identified the significant role of climate seasonality in maintaining the species richness patterns of *Rhododendron* and higher plants. A negative relationship between species richness and climatic seasonality has previously been reported (Gao & Liu, 2018; Kristiansen et al., 2011; Liu et al., 2017; Panda et al., 2017; Pandey et al., 2020; Shrestha et al., 2017), and our study supports the climate seasonality hypothesis. The MAT in China is between –25 and 25°C, whereas the temperature range is 11–59.7°C, there is high fluctuation in temperature, where the northern regions experience lower extreme temperature than do the southern regions. This temperature extremity might be the reason for the restricted distribution of gymnosperms in the northern part of China. The failure of gymnosperms to adapt and their incapability to migrate from south to north is in accordance with our findings, which support the tropical niche conservatism theory (Romdal, Araújo, & Rahbek, 2013). This climatic seasonality in the southwestern part of country has created a stable climate for the species to survive in harsh conditions (Panda et al., 2017). Moreover, Dakhil et al. (2019) reported the significant role of the climatic stability of the warmest quarter during the Quaternary Period (until now). This climatic stability is an ecological indicator of the range stability of cold temperate conifers in the high elevation regions of southwestern China, including the eastern part of the Tibetan Plateau (Liao et al., 2020). Climatic variability and unsystematic changes in daily maximum and minimum temperature increase the level of tolerance of an organism by altering the thermal environment that organisms experience, thus enabling them to become geographically widespread.

Our findings also imply that energy–water was responsible for the distribution pattern of all species and endemic species of gymnosperms in China. Similar findings were reported in the studies conducted in China and the Himalayan region to explain the species richness patterns of gymnosperms (Lü et al., 2018; Pandey et al., 2020; Subedi et al., 2019; Yang, 2015), Gesneriaceae (Liu et al., 2017), and *Rhododendron* (Shrestha et al., 2017). Our study also supports the findings of Lü et al. (2018), where energy (MAT) and water (MAP) were significant variables in explaining the richness patterns of all species of gymnosperms in China. In this study, we hypothesized that species richness increases with increasing energy and water availability. The energy–water variable was the second‐best predictor of species richness patterns in all species and endemic species, which is in accordance with previous studies conducted on plants (Kreft & Jetz, 2007; Pandey et al., 2020; Rahbek, 2005). Photosynthesis in plants is always favored by available energy and moisture and promotes species richness by influencing all physiological processes (Adler & Levine, 2007; Bhattarai & Vetaas, 2003; Currie et al., 2004; Hawkins et al., 2003). High precipitation and temperature implies that the energy–water available to the plant is high, thus reducing stress in gymnosperms. These findings arguably reflect that energy–water is important in determining the species diversity in tropical regions (Hawkins et al., 2003). Feng, Mao, Sandel, Swenson, and Svenning (2016) found that the richness patterns of endemic plant species are determined by the current precipitation. The response of energy–water to endemism is profoundly explained in variation partitioning, which is supported by the findings of McKenzie and Rosenberg (2001). The energy–water variables used in this study are derived from the temperature and precipitation records of the area, which may explain why highest endemic species richness occurs in the Hengduan Mountains and Qinling‐Daba Mountains; these areas are rich in both available energy and water. It was noticed that the endemic richness was confined to southwest China, which is characterized by a tropical climate with abundant energy and water. Water‐related variables are the best predictors for plants that are distributed from tropical to sub‐alpine regions, provided that energy is abundant (Hawkins et al., 2003). Rainfall is higher in the southwest than in the north of China. Therefore, southwest China has the highest diversity of all species and endemic species of gymnosperms. Thus, it can be predicted that, because of climate change, endemic species will be profoundly affected. These species are confined to a specific area and might face a high risk of extinction due to global warming and changes in climatic conditions over time.

The southwest region and Taiwan, China, features topographical variations caused by elevational differentiation, which collectively results in a variety of hydrothermal conditions and habitats (Li et al., 2009; Liu et al., 2017; Lü et al., 2018), and thus, the highest richness values for all, endemic, and nonendemic species. In this study, we used the elevation range as the main predictor of habitat heterogeneity, which is arguably the best indicator of topographic variation. This finding is also supported by Shrestha et al. (2017), Liu et al. (2017), and Lü et al. (2018). Habitat heterogeneity is considered to be the most critical factor that shapes the distribution patterns of organisms; habitat variation allows species to coexist in the locality by creating a steep climate and habitat differentiation in small areas, thus making a microhabitat for species to flourish (Kreft & Jetz, 2007; Pausas & Austin, 2001; Tamme et al., 2010). In addition, allopatric speciation might have impacted the growth of gymnosperms in isolated mountains, flourishing the growth of one species while restricting that of others. The Qinghai–Tibetan Plateau uplift, which has profoundly changed the geomorphology of the Chinese mainland, has created mountains, gullies, and canyons, forming a huge difference in the elevational gradients of the mountains. Quaternary ice sheet invasion prompted gymnosperms to migrate from high to low altitudes, while the periodic effects of climate warming cause the plants to migrate back to high‐altitude areas (Calatayud et al., 2016). Plant groups that are unable to migrate could only adapt to differentiation in low‐ and middle‐altitude environments, therefore causing the high endemic species richness of gymnosperms. Habitat heterogeneity also provides suitable refuges from adverse climatic conditions; for example, glaciation may influence the diversification of species through habitat isolation and limited migration (Calatayud et al., 2016; Liao et al., 2020). Moreover, highly heterogeneous tropical and subtropical mountains might act as cradles of biodiversity and thus are dominant in terms of species richness and concentrations of narrow endemic species (Liao et al., 2020).

Human‐influenced variables showed the least involvement in the species richness patterns of gymnosperms. Therefore, it can be predicted that human disturbance has had little impact on the richness patterns of gymnosperms in China. This might be because of the effectiveness of the conservation policy implemented by the Chinese Government via the Natural Forest Conservation Program to conserve the natural heritage of the country (Viña, McConnell, Yang, Xu, & Liu, 2016). However, there are studies that have reported cases of human disturbance in China (Xu et al., 2019), and several instances of disturbances causing the depletion of species have also been reported in other countries (Potapov et al., 2017; Stevens et al., 2017). In this human‐dominated world, biodiversity is facing growing pressure due to habitat degradation, habitat fragmentation, land use change, climate change, forest exploitation, and pollution (Newbold et al., 2015). Because of these anthropogenic impacts, it is more likely that species with narrow distribution ranges (endemic species) are more likely to become extinct than are species with wide distributional ranges (Xu et al., 2019). In the southwestern region of China, human disturbances, such as hydropower station construction, road construction, grazing, and drug digging, are gradually increasing. Balancing the contradiction between conservation and development is of great significance for maintaining the highly enriched endemic gymnosperm diversity in this region. Therefore, disturbance scenarios cannot be overlooked and further research is required to verify these findings. Relevant protection and development technologies also require further study. Currently, our knowledge of anthropogenic threats is still limited; thus, long‐term monitoring and applied research are needed in this montane biodiversity hotspot to provide more valuable insights for biodiversity conservation in the context of global change.

## 5 CONCLUSION

This study explored the spatial species richness patterns of Chinese gymnosperms and provides a possible explanation for the results based on multiple environmental and human‐induced factors. The species distribution of gymnosperms is high in the southwest and Taiwan regions, while the north and Tibetan regions have the lowest distribution of gymnosperm species. Climatic seasonality is a potent variable that represents the potential distribution and species richness variation of gymnosperms in China. Energy–water and habitat heterogeneity partly explain the richness patterns of gymnosperms. Collectively, gymnosperm richness in China is highly associated with climatic seasonality, energy–water, and habitat heterogeneity, but less influenced by human‐induced effects.

## CONFLICT OF INTEREST

There are no conflicts of interest to declare.

## AUTHOR CONTRIBUTION

**Bikram Pandey:** Conceptualization (lead); Data curation (lead); Formal analysis (lead); Methodology (lead); Writing‐original draft (lead); Writing‐review & editing (lead). **Janak Raj Khatiwada:** Formal analysis (supporting); Writing‐original draft (supporting); Writing‐review & editing (supporting). **Lin Zhang:** Conceptualization (supporting); Methodology (supporting); Writing‐review & editing (supporting). **Kaiwen Pan:** Conceptualization (supporting); Funding acquisition (lead); Investigation (supporting); Project administration (lead); Supervision (lead); Writing‐review & editing (supporting). **Mohammed Dakhil:** Formal analysis (supporting); Methodology (supporting); Writing‐original draft (supporting); Writing‐review & editing (supporting). **Qinli Xiong:** Writing‐review & editing (supporting). **Ram Kailash Prasad Yadav:** Methodology (supporting); Writing‐review & editing (supporting). **Mohan Siwakoti:** Methodology (supporting); Writing‐review & editing (supporting). **Akash Tariq:** Writing‐original draft (supporting); Writing‐review & editing (supporting). **Olusanya Abiodun Olatunji:** Writing‐original draft (supporting); Writing‐review & editing (supporting). **Meta Francis Justine:** Resources (supporting); Writing‐review & editing (supporting). **Xiaogang Wu:** Data curation (supporting); Writing‐review & editing (supporting). **Xiaomin Sun:** Resources (supporting); Writing‐review & editing (supporting). **Ziyan Liao:** Formal analysis (supporting); Writing‐review & editing (supporting). **Zebene Tadesse Negesse:** Resources (supporting); Writing‐review & editing (supporting).

## Supporting information

Supplementary Material

## Data Availability

All the predictor variables were extracted from online database and are cited in the manuscript with an accessible URL. Data with species richness information is archived in Dryad Digital Repository: https://doi.org/10.5061/dryad.0p2ngf1xz.
