# Supplementary material for: Energy–water and seasonal variations in climate underlie the spatial distribution patterns of gymnosperm species richness in China
Source: Ecol Evol. 2020 Aug 8;10(17):9474–85. doi: 10.1002/ece3.6639 (PMC7487259; doi:10.1002/ece3.6639)
Supplement: Supplementary file 1 — Supplementary Material [file ECE3-10-9474-s001.docx]

# SUPPORTING INFORMATION

Table A1: Results of principal components analysis using energy-water, habitat heterogeneity, climatic seasonality and human-induced sets of variables.

| **Energy**-**water** | **PC1** | **PC2** | **PC3** |
| --- | --- | --- | --- |
| MAT | 0.149 | 0.257 | 0.530 |
| PET | 0.128 | 0.424 | 0.250 |
| MAP | 0.149 | –0.283 | 0.152 |
| AET | 0.145 | –0.280 | 1.073 |
| MAT^2^ | 0.151 | 0.019 | –1.153 |
| PET^2^ | 0.130 | 0.417 | –0.137 |
| MAP^2^ | 0.143 | –0.238 | –1.144 |
| AET^2^ | 0.155 | –0.214 | 0.427 |
| Proportion of variance (%) | 75.3 | 18.4 | 2.9 |
| Cumulative proportion (%) | 75.3 | 93.7 | 96.6 |
|  | | | |
| **Habitat heterogeneity** | **PC1** | **PC2** | **PC3** |
| MElv | 0.365 | –0.07 | –0.339 |
| VEL | –0.314 | 0.101 | –0.391 |
| ELR | 0.101 | 0.506 | 0.044 |
| MElv ^2^ | 0.353 | –0.069 | –0.358 |
| VEL^2^ | –0.286 | 0.101 | –0.423 |
| ELR^2^ | 0.118 | 0.502 | 0.023 |
| Proportion of variance (%) | 36.0 | 31 | 28.9 |
| Cumulative proportion (%) | 36.0 | 67 | 95.9 |
|  | | | |
| **Climatic seasonality** | **PC1** | **PC2** | **PC3** |
| TES | 0.252 | –0.068 | 0.607 |
| PES | 0.154 | 0.478 | –0.184 |
| ART | 0.232 | –0.249 | –0.530 |
| TES^2^ | 0.253 | –0.080 | 0.598 |
| PES^2^ | 0.159 | 0.473 | –0.161 |
| ART^2^ | 0.237 | –0.225 | –0.538 |
| Proportion of variance (%) | 58.2 | 28.9 | 12.3 |
| Cumulative proportion (%) | 58.2 | 87.1 | 99.4 |
|  | | | |
| **Human influence** | **PC1** | **PC2** | **PC3** |
| HFI | 0.260 | –1.264 | 1.282 |
| HII | 0.260 | –1.288 | –1.254 |
| HFI^2^ | 0.258 | 1.299 | 1.640 |
| HII^2^ | 0.258 | 1.274 | –1.669 |
| Proportion of variance (%) | 93.2 | 3.8 | 2.9 |
| Cumulative proportion (%) | 93.2 | 97 | 99.9 |

MAT, mean annual temperature; PET, potential evapotranspiration; MAP, mean annual precipitation; AET, actual evapotranspiration; MElv, mean elevation; ELR, range of elevation; VEL, coefficient of variation in elevation; TES, temperature seasonality; PES, precipitation seasonality; ART, annual range of temperature; HII, human influence index; HFI, human footprint index.

Table A2: Variance Inflation Factor (VIFs) of all individual variables used to explain the species richness pattern of gymnosperms. AIC is the Akaike’s information criterion value of a model.

|  | Variables | VIF | AIC |
| --- | --- | --- | --- |
| All Species | CS1 | 1.135 | 9339.0 |
|  | CS2 | 1.386 |  |
|  | EW2 | 1.121 |  |
|  | HH1 | 1.543 |  |
|  | EW3 | 1.096 |  |
|  | HE2 | 1.136 |  |
| Endemic | CS1 | 1.057 | 5851.1 |
|  | EW3 | 1.098 |  |
|  | CS2 | 1.037 |  |
|  | EW2 | 1.129 |  |
| Non endemic | CS1 | 1.073 | 5855.6 |
|  | HH1 | 1.006 |  |
|  | HH2 | 1.078 |  |
|  | HE3 | 1.007 |  |

EW, CS, HH and HE refer to variables based on the first three axes of the PCA using energy-water, climatic seasonality, habitat heterogeneity, and human influence effect variables, respectively. See Table A1 for the individual contribution of each explanatory variable to each principal component.


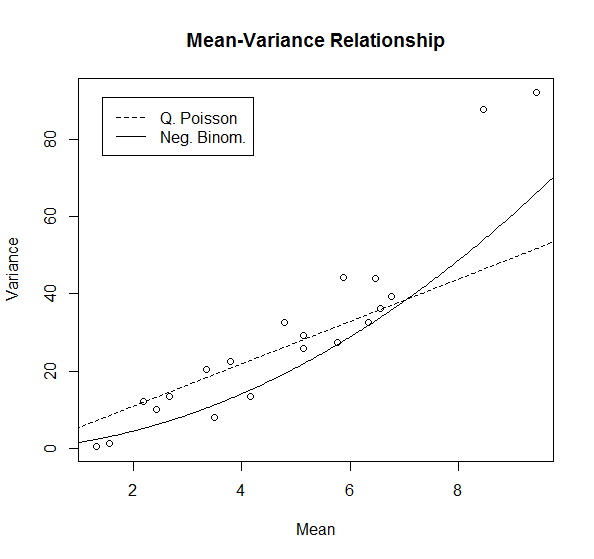


Figure A3: Estimated mean–variance relationship for gymnosperm species in China. The dashed line is the quasi-Poisson error distribution model and the solid line is the negative binomial regression (NBR) model (dispersion parameters of quasi-Poisson error distribution and NBR models were 5.3193 and 1.6283, respectively).


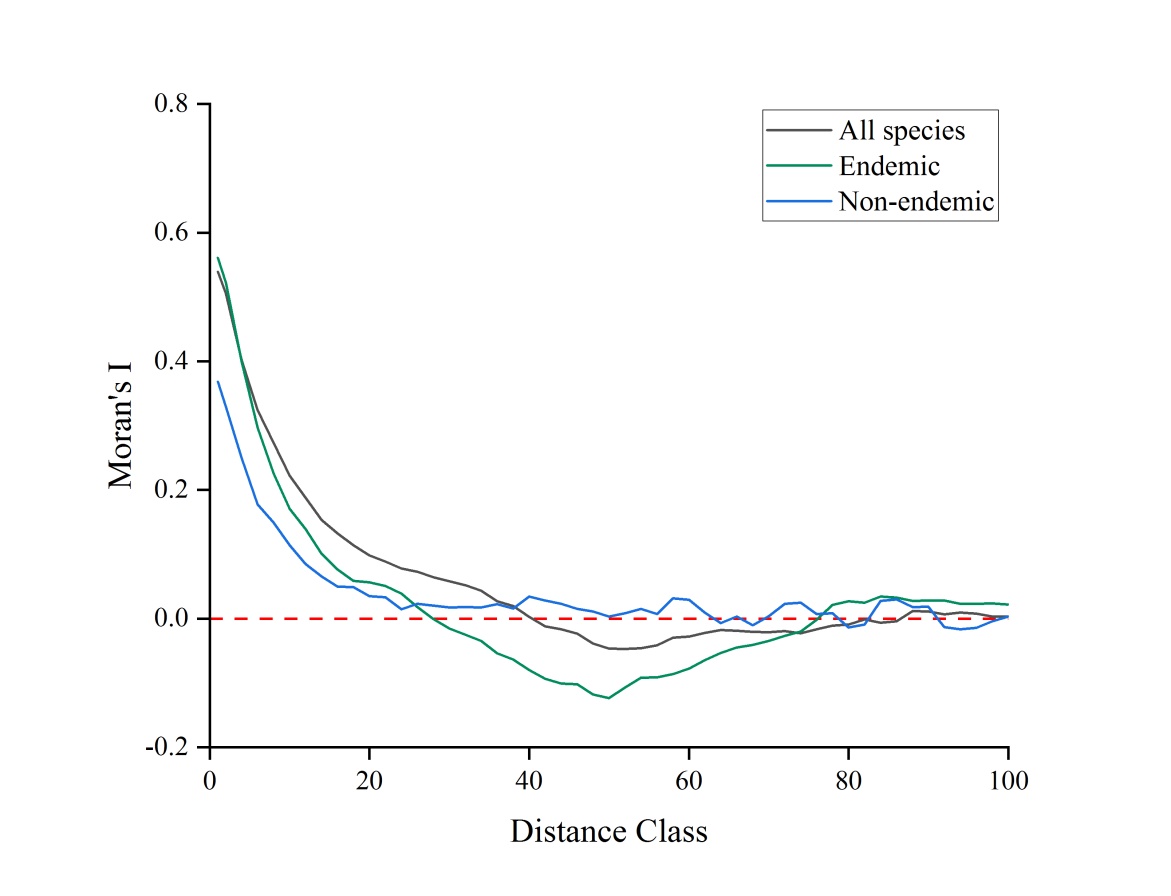


Figure A4: Moran’s index correlograms for gymnosperm richness and residual autocorrelation.

Table A5: General statistical summary of the species richness of all, endemic and non-endemic species of gymnosperms

| **Variables** | **Total grid** | **Mean richness per grid** | **Minimum** | **Maximum** | **Mean** | **SE** | **SD** | **Skewedness** | **Kurtosis** |
| --- | --- | --- | --- | --- | --- | --- | --- | --- | --- |
| **All species** | 1837 | 4.78 | 1 | 49 | 4.78 | 0.14 | 5.84 | 2.58 | 8.66 |
| **Endemic** | 1197 | 4.18 | 1 | 39 | 4.18 | 0.13 | 4.36 | 2.37 | 8.03 |
| **Non-endemic** | 1496 | 2.60 | 1 | 21 | 2.6 | 0.06 | 2.62 | 2.74 | 9.85 |

Table A6: Results of variation partitioning showing the percentage contributions of predictor variables to determine the species richness of gymnosperms. The lowercase letter denotes the individual contribution (adjusted *R*^2^) following the labels displayed in Figure 2a (main text).

| **Individual contribution (%)** | **Species richness** | | |
| --- | --- | --- | --- |
|  | **All species** | **Endemic** | **Non-endemic** |
| [a] | 9.64 | 6.82 | 1.91 |
| [b] | 38.16 | 43.18 | 37.16 |
| [c] | 0.04 | 9.24 | 18.89 |
| [d] | 4.44 | 0.80 | 3.28 |
| [e] | 11.24 | 12.03 | 0.29 |
| [f] | 7.36 | 0.42 | 8.09 |
| [g] | 0.05 | 2.71 | –0.30 |
| [h] | 0.16 | –5.69 | 0.05 |
| [i] | 0.18 | –1.47 | 0.56 |
| [j] | –0.07 | –0.61 | 0.03 |
| [k] | 2.03 | 4.25 | –0.08 |
| [l] | 3.35 | 3.29 | 0.04 |
| [m] | –0.38 | 1.13 | 5.08 |
| [n] | –0.06 | 0.23 | 3.07 |
| [o] | 7.07 | 6.05 | 0.17 |
| [p] = residuals | 16.80 | 17.63 | 21.76 |
|  |  |  |  |
| Total energy-water set [aeghklno] | 33.47 | 29.68 | 5.16 |
| Total climatic seasonality set [befiklmo] | 69.00 | 68.86 | 51.30 |
| Total habitat–heterogeneity set [cfgjlmno] | 17.36 | 22.46 | 35.06 |
| Total human effect set [dhijkmno] | 13.36 | 4.69 | 12.16 |
